# The Use of Selected Strains of Probiotic Lactic Acid Bacteria to Eliminate Aflatoxin M1 From Brined White Cheese

**DOI:** 10.1155/ijfo/4013930

**Published:** 2026-06-01

**Authors:** Murad A. Al-Holy, Hiba A. Al-Masri, Amin N. Olaimat, Hamzah Al-Qadiri, Maisa Monjed, Mutamed Ayyash, Mahmoud Abughoush, Ma’mon M. Gharaibeh

**Affiliations:** ^1^ Department of Clinical Nutrition and Dietetics Faculty of Applied Medical Sciences The Hashemite University Zarqa Jordan hu.edu.jo; ^2^ Department of Nutrition and Food Technology School of Agriculture University of Jordan Amman Jordan ju.edu.jo; ^3^ Department of Food Science United Arab Emirates University Al Ain UAE uaeu.ac.ae; ^4^ Science of Nutrition and Dietetics Program College of Pharmacy Al Ain University Abu Dhabi UAE aau.ac.ae; ^5^ Food Lab Division Jordan Food and Drug Administration Amman Jordan

**Keywords:** bio-removal, brined cheese, dairy products, detoxification, mycotoxins

## Abstract

This study aimed to investigate the ability of selected strains of probiotic lactic acid bacteria (LAB) to remove aflatoxin M1 (AFM1) from brined white cheese (BWC) immersed in a 7.5% brine solution. AFM1 was added to the milk used to prepare the cheese. The final concentration of AFM1 was ca. 125.0 ng/kg. Thereafter, potential probiotics LAB (*Lactobacillus acidophilus*, *Lactobacillus delbrueckii* subsp. delbrueckii, *Lacticaseibacillus paracasei* subsp. paracasei, *Limosilactobacillus reuteri*, and *Lactobacillus gasseri*) were delivered into BWC, by inoculating each of the bacteria individually into the brine at levels that ranged between ca. 8.0 and 9.0 log CFU/g. The detoxification activity of the probiotics was monitored after 0, 24, and 72 h of incubation at 4°C, 10°C, or 24°C. The pH, water activity (a_w_), and salt concentrations of inoculated BWC were examined initially and after 72 h. The probiotic LAB survived in BWC, at the various storage temperatures, where the counts remained at levels > 7.0 log CFU/g after 72 h. The detoxification of AFM1 by probiotics was temperature, time, and probiotic strain dependent. *L. paracasei* subsp. paracasei exerted the most effective detoxification activity at 24°C after 72 h of incubation, where it resulted in reducing AFM1 by almost six times (from the initial level of *ca.* 125.0 ng/kg to 22.2 ng/kg). At 10°C, *L. paracasei* followed by *L. reuteri* resulted in significantly lower (*p* < 0.05) AFM1 concentrations than the other probiotics. Potential probiotic LAB such as *L. paracasei* subsp. paracasei and *L. reuteri* (SS730, S3608, CF2) could be used effectively to eliminate considerable amounts of AFM1 contamination n BWC.

## 1. Introduction

BWC is the most popular cheese manufactured and consumed in the Middle East and North African countries [1–3]. It is usually produced from cows’ milk by rennet coagulation and characterized by having a soft to semi‐hard texture [4]. Traditionally, BWC is consumed either after ripening at room temperature in 5%–20% brine solution for a duration that could last between 2 and 12 months or could be consumed fresh [1]. BWC is a perishable food that can provide an ideal habitat for the growth of foodborne pathogens because of its high‐ a_w_ (0.94–0.96), moderate fat content (10.4%), relatively high pH value (5.2–6.1), high protein (21.1%), and the absence of competitive starter culture [5, 6]. The presence of foodborne pathogens or their toxins in the food system continues to pose a remarkable challenge to the food industry [7]. Some of the main causes of food poisoning are outbreaks related to food contamination with enterotoxin‐producing microorganisms such as *Staphylococcus aureus* and mycotoxins production by some fungal species [3, 8, 9].

A critical issue in food safety is that some mold species produce mycotoxins, which pose serious health risks and are transferred through the food chain. Mycotoxins are extremely toxic secondary metabolites produced by mold genera, such as *Aspergillus*, *Penicillium*, and *Fusarium* [10, 11]. Aflatoxins are the most carcinogenic mycotoxin secondary metabolites produced mainly by strains of *A. flavus* and *A. parasiticus* [11]. AFM1 is the hydroxylated metabolite of aflatoxin B1 (AFB1) and can be found in milk and other dairy products when lactating animals are fed with contaminated feedstuff [12]. Once in milk, AFM1 is not degraded and can withstand different industrial heat treatment protocols [13]. Hence, AFM1 contamination persists as a serious problem in produced milk and all its derived dairy products, including cheese [13]. AFM1 is concentrated during cheese making but is not uniformly distributed between curd and whey, with higher levels found in the curd due to a stronger association with casein, and AFM1 concentration is often 3 to 5 times higher in the cheese compared with the milk used for its production [14]. The acceptable limit set by the European Union for AFM1 concentration in milk is 50 and 25 ng/kg for infant milk products [15]. In comparison, the maximum level established by the United States Food and Drug Administration (FDA) is 500 ng/kg in milk [16].

Because of aflatoxins’ unique stability under different processing and storage conditions of food [17], different biological, chemical, and physical methods have been tested to reduce aflatoxin in the food and feed materials [18]. Food composition, decontamination conditions, and decontamination techniques are factors that affect the effectiveness of aflatoxin removal [19]. Physical treatments have proven effective in reducing aflatoxin concentrations, including cold plasma, electrolyzed water, electron beam, gamma irradiation, microwave heating, UV, and pulsed light [19, 20]. On the other hand, organic acids, such as citric, lactic, tartaric, and propionic acids, and hydrochloric acid, as well as fungicides, are used as chemical treatments to degrade aflatoxins [19, 20]. Notwithstanding, removal of AFM1 from milk with chemical and physical agents has some disadvantages, such as insufficiency of toxin removal with high cost and loss of nutritional value [18]. This highlighted the use of biological treatments, such as bacteria, yeasts, molds, and algae, to remove aflatoxin through biological competition for space and nutrients, interactions, and antibiosis [21]. Most of the candidate microorganisms are probiotics, which are “generally recognized as safe” (GRAS), and already used in food production, such as LAB [22]. Most probiotics belong to the LAB family [23]. Probiotics may offer a promising approach to controlling pathogenic bacteria in the food industry, thereby reducing the potential of antimicrobial resistance associated with the intensive use of chemical preservatives [24]. Some LAB are capable of producing lactic acid and other metabolites with specific antagonistic and antibacterial activities, such as bacteriocins and antifungal compounds, which have considerable potential to inhibit various types of microorganisms [25]. Several studies explored the potential of protective LAB cultures to control pathogens in cheese [26]. The antimicrobial activity of probiotic LAB emanates from their secreted metabolites (e.g., organic acids, bacteriocins, and biosurfactants) and competition with foodborne pathogens for nutrients [27]. However, only few studies investigated the potency of using probiotic LAB to decontaminate aflatoxins in dairy products [28, 29]. Hence, the present study aimed to explore the capability of selected strains of probiotic LAB (*Lactobacillus. acidophilus* DSMZ 9126, *Lactobacillus delbrueckii* subsp. delbrueckii DSMZ 20074, *Lacticaseibacillus paracasei* subsp. paracasei DSMZ 202071, *Limosilactobacillus reuteri* (SS730, S3608, CF2), and *Lactobacillus gasseri* DSMZ 20243) to remove AFM1 from BWC immersed into 7.5% brine solution stored at 4°C, 10°C, or 24°C for 30 days storage duration.

## 2. Material and Methods

### 2.1. Processing of White Cheese

BWC was processed as per the method depicted by Al‐Nabulsi et al. [1], in which pasteurized full‐fat cow’s milk was used to produce the cheese. Fresh milk was pasteurized by heating at 72°C for 15 s, then cooling to 36°C–37°C. Thereafter, the milk was coagulated into a semisolid curd by rennet enzyme (Chris Hanseb, Hoshlom, Denmark). The renneting process lasted for 40 min, restricting much of the fat. Afterwards, whey protein was drained for 20 min and stainless steel plate was used to press the curd at room temperature for 60 min. The resulting pressed curd was cut into cubes with approximate weight of 10–15 g, transferred into 7.5% (w/v) sterile brine, and then stored at 4°C.

### 2.2. Bacterial Strains

The bacterial strains used in the current study were obtained from the Food Microbiology Laboratory, the Hashemite University (Zarqa‐Jordan). Seven strains from five different species of LAB, namely, *L. acidophilus* DSMZ 9126, *L. delbrueckii* subsp. delbrueckii DSMZ 20074, *L. paracasei* subsp. paracasei DSMZ 202071, *L. gasseri* DSMZ 20243, and three strains of *L. reuteri* (SS730, S3608, CF2) were used. Frozen stocks of all cultures were maintained at −20°C in 15% glycerol. After thawing, a loopful of each culture of probiotic LAB was streaked onto de Man, Rogosa, and Sharpe MRS) agar (Oxoid Ltd., Basingstoke, England) and then incubated at 37°C for 24 h under anaerobic conditions using a CO_2_‐generating kit (AnaeroGen, Oxoid Ltd., Basingstoke, England). Then, a single colony of each of the probiotic LAB strain was activated by transferring into MRS broth. The strains were sub‐cultured twice in MRS broth under anaerobic conditions for 24 h at 37°C. Cultures of LAB strains were harvested by centrifugation for 18 min at 1500 rpm and washed again. The resulting pellet was subsequently collected in 10 mL of 0.1% peptone water to reach the desired concentration of about 9–10 log CFU/mL. To prepare 10 mL bacterial cocktail of *L. reuteri*, 3.3 mL of each *L. reuteri* strain was placed into sterile tube. The resulting *L. reuteri* cocktail was mixed vigorously and centrifuged at 1500 rpm for 18 min (Nüve, Istanbul, Turkey). The supernatant was discarded, and the settling pellet was washed using 10 mL of 0.1% peptone water (Oxoid Ltd., Basingstoke, England). The pellet–peptone water mixture was mixed vehemently by means of a vortex mixer. The *L. reuteri* cultures were subject to washing and centrifugation again, and the collected pellets were mixed into 10 mL of 0.1% peptone water to yield 9–10 log CFU/mL.

### 2.3. Detoxification of AFM1 in BWC Using Different Probiotic LAB

#### 2.3.1. Contamination of the White Cheese With AFM1

The procedure described by Adácsi et al. [30] was used to inoculate milk with AFM1, with some modifications. Exactly 10 *μ*g of AFM1 (Sigma, St. Louis, MO, United States) was dissolved in acetonitrile under a chemical cabinet using a chemical mask and nitrile gloves. AFM1 was added to fresh milk at a level of 1.0 *μ*g/L. Thereafter, the milk was subject to pasteurization for 15 s at 72°C. The milk was cooled to 40°C, coagulated with rennet, and white cheese was prepared according to the procedure described previously. The cheese was cut into 2 × 5 × 5 cm piece. The cheese samples (150 g each) were placed into sterile plastic bags, and 200 mL of sterile 7.5% brine solution was added to each bag. The initial concentration of AFM1 in BWC before inoculation of the probiotic LAB was ca. 125.0 ng/kg of BWC.

#### 2.3.2. Detoxification of AFM1 Using Probiotic Bacteria

Five different types of probiotic bacteria (*L. acidophilus* DSMZ 9126, *L. delbrueckii* subsp. delbrueckii DSMZ 20074, *L. paracasei* subsp. paracasei, *L. reuteri* [SS730, S3608, and CF2], and *L. gasseri* DSMZ20243) were suspended into 20% glycerol and inoculated individually into the brine solution (7.5%) surrounding the white cheese in the bags at the level 10^8^–10^9^ CFU/mL. The BWC that was not inoculated with any of the probiotic LAB was considered a control and used for comparison purposes. Different samples of inoculated BWC were stored at three different temperatures (4°C, 10°C, and 24°C) for 72 h and the concentrations of AFM1 were determined after incubating the samples for 0, 24, and 72 h at the different corresponding storage temperatures. For the determination of probiotic LAB numbers at the corresponding time intervals, 100 *μ*L of appropriate dilutions were placed onto the surface of MRS (All LAB) and Rogosa (for *L. reuteri*) (Oxoid Ltd., Basingstoke, England) and incubated under anaerobic conditions using a CO_2_ generating kit (AnaeroGen, Oxoid) for 48 h at 37°C.

#### 2.3.3. Determination of Residual AFM1 in BWC

The residual concentrations of AFM1 in cheese were determined using a high‐sensitivity 96‐well direct competitive enzyme‐linked immunosorbent assay (ELISA) that quantitatively determines AFM1 presence (5–100 ppt) (AgraQuant, Romers Labs, Getzersdorf, Austria). Five working standards, 5, 10, 50, 70 and 100 ng/L of milk were prepared from AFM1 (Sigma, St. Louis, MO, United States). Cheese samples were prepared for AFM1 analysis by grating 2.0 g of cheese samples into 50 mL flasks and then 20 mL of preheated (50°C) sterile deionized water was added. The samples were extracted by shaking for 30 min in a rotary shaker set at 250 rpm at room temperature. Thereafter, the slurries were filtered using Whitman #1 filter paper and the resulting filtrates were ready for testing. The ELISA protocol specified by the manufacturer was carefully followed. The absorbance of each well was read within 10 min after the addition of the stop solution at 450 nm with a microwell reader. The residual concentrations of AFM1 were expressed as ng/kg of cheese.

### 2.4. Determination of a_w_, pH, and Salt Content of BWC

The a_w_ of BWC stored at the various storage temperatures was measured using an a_w_ meter (Novasina AG, Labmasters aw, Lachen, Switzerland). Nearly, 2‐g samples from cross‐sectioned BWC were used to determine a_w_ at room temperature initially (0 h) and after 72 h of storage at the various temperatures. Likewise, the pH values of cheese samples were measured using a pH meter (Adwa pH meter, 1000 CE, Adwa, Romania), initially (0 h) and after 72 h of incubation at the corresponding incubation temperatures (4°C, 10°C, and 24°C). Cheese slurries were prepared by grinding ca. 5.0 g of the cheese sample with 10 mL of distilled water. The electrode of the pH meter was dipped into the cheese slurry and the readings were taken for triplicate samples after readings became completely stable. On the other hand, the salt content of cheese samples was determined according to the Association of Official Analytical Chemists (AOAC), AOAC 983.14 method [31]. The method involves determining the chloride content by titration with 0.05 N silver nitrate (Carlo Erba, Crloerba, France). A few drops of potassium chromate (0.5 N) (Alpha Chemika, Mumbai, India) was used as an indicator. Approximately 3.0 g of pummeled BWC sample was weighed. Thereafter, 30 mL of distilled water was added and mixed the pummeled cheese at 55°C. Then 2 mL of HNO_3_ (in 0.5 mL aliquots) (Carlo Erba, Crloerba, France) was added and the mixture was titrated with AgNO_3_. Triplicate samples of cheese were analyzed and the % salt content was determined as per the following equation:

%NaCl=V×N×5.84/sample weight

### 2.5. Statistical Analysis

The Statistical Package for the Social Sciences (SPSS), Version 25.0 (IBM Corp., Armonk, NY) for quantitative data analysis, was used to statistically analyze the results. In the current study, each value is the average of three independent trials. To examine the effect of each treatment factor, a one‐way analysis of variance (ANOVA) test was performed. Values are given as means ± standard deviations. The statistical significance was determined using Tukey’s HSD test at a *p* value of 0.05 among various treatments or storage times.

## 3. Results

### 3.1. Initial and Final Salt Content and a_w_ Values of BWC Stored at Different Temperatures

Table 1 shows the initial (0 h) and final (72 h) salt concentrations and a_w_ values of BWC immersed in 7.5% brine solution. In general, as the storage temperature increased the percentage of salt content in BWC increased. A significant increase (*p* < 0.05) in salt concentration was observed in BWC at 10°C and 24°C. The initial salt concentrations in the BWC were 3.23%, 3.08%, and 2.37%, and after 72 h of storage, the salt content increased to 3.30%, 3.69%, and 3.72% at 4°C, 10°C, and 24°C, respectively. On the other hand, a slight reduction in a_w_ was noticed when the BWCs were kept in solution at 4°C and 24°C, but this drop was not significantly different (*p* > 0.05). The lowest a_w_ was observed for the BWC incubated at 24°C, where the a_w_ dropped from an initial value of 0.96–0.95.

**Table 1:** Initial and final salt content and a_w_ values of BWC preserved in 7.5% brine and stored at various temperatures for 72 h.

Temperature (°C)	Initial salt (0 h)^a^	Final salt (72 h)^a^	Initial a_w_ (0 h)^a^	Final a_w_ (72 h)^a^
4	3.23 ± 0.04^aA^	3.30 ± 0.28^aB^	0.97 ± 0.00^aA^	0.96 ± 0.00^aA^
10	3.18 ± 0.03^aA^	3.69 ± 0.02^bA^	0.97 ± 0.00^aA^	0.97 ± 0.01^aA^
24	3.27 ± 0.04^aA^	3.72 ± 0.03^bA^	0.96 ± 0.00^aA^	0.95 ± 0.01^aA^

^a^Data represent means ± SD of three independent trials. Means of salt or a_w_ in the same column with different uppercase letters are significantly different (*p* < 0.05). Means salt or a_w_ in the same row with different lowercase letters are significantly different (*p* < 0.05).

### 3.2. Initial and Final pH Values of BWC Contaminated With AFM1 and Inoculated With Different Strains of Probiotic Lactic Acid Bacteria

Table 2 presents the initial and final pH values in BWC contaminated with AFM1 and different strains of probiotic LAB (*L. acidophilus* DSMZ 9126, *L. delbrueckii* subsp. delbrueckii DSMZ 20074, *L. paracasei* subsp. paracasei DSMZ 202071, *L. reuteri* [SS730, S3608, CF2], and *L. gasseri* DSMZ 20243) and stored for 72 h at 4°C, 10°C, or 24°C. In the control (BWC without any probiotic LAB), no significant reduction was accrued at 4°C, 10°C. However, after 72 h of incubation at 24°C, a significant drop in the pH value of the control took place. Notwithstanding, it was evident that inoculated probiotics lead to a significant drop in the pH values of BWC after 72 h of incubation at 24°C compared with the control.

**Table 2:** Initial and final pH values of BWC (7.5% brine) contaminated with AFM1and inoculated with different strains of probiotic Lactobacilli and stored at various storage temperatures for 72 h.

Temperature (°C)	Probiotic type^a^	Initial pH (0 h)^b^	Final pH (72 h)^b^
4	Control	6.62 ± 0.01^aA^	6.45 ± 0.04^aA^
	1	6.57 ± 0.02^aA^	6.44 ± 0.05^aA^
	2	6.57 ± 0.02^aA^	6.57 ± 0.03^aA^
	3	6.67 ± 0.06^aA^	6.33 ± 0.01^bA^
	4	6.59 ± 0.03^aA^	6.48 ± 0.02^aA^
	5	6.53 ± 0.04^aA^	6.42 ± 0.04^aA^
10	Control	6.46 ± 0.01^aA^	6.44 ± 0.11^aA^
	1	6.56 ± 0.01^aA^	6.41 ± 0.03^aA^
	2	6.64 ± 0.01^aA^	6.52 ± 0.02^aA^
	3	6.62 ± 0.03^aA^	6.48 ± 0.16^aA^
	4	6.52 ± 0.01^aA^	6.36 ± 0.09^bA^
	5	6.55 ± 0.06^aA^	6.42 ± 0.14^aA^
24	Control	6.57 ± 0.01^aA^	6.38 ± 0.04^bA^
	1	6.54 ± 0.01^aA^	6.20 ± 0.04^bB^
	2	6.52 ± 0.02^aA^	6.28 ± 0.05^bB^
	3	6.56 ± 0.05^aA^	6.24 ± 0.02^bB^
	4	6.64 ± 0.01^aA^	6.14 ± 0.03^bB^
	5	6.55 ± 0.01^aA^	6.21 ± 0.02^bB^

*Note:* Control: un‐inoculated with any probiotic.

^a^1: *Lactobacillus acidophilus* DSMZ 9126; 2: *Lactobacillus delbrueckii* subsp. delbrueckii DSMZ 20074; 3: *Lacticaseibacillus paracasei* subsp. paracasei DSMZ 202071; 4: *Limosilactobacillus reuteri*(SS730, S3608, CF2); 5: *Lactobacillus gasseri* DSMZ 20243.

^b^Data represent means ± SD of three independent trials. Means in the same column with different capital letters are significantly different (*p* < 0.05). Means in the same row with different small letters are significantly different (*p* < 0.05).

### 3.3. Growth Pattern of Different Types of Probiotic LAB in BWC Stored at Different Temperatures

**Figure 1:**
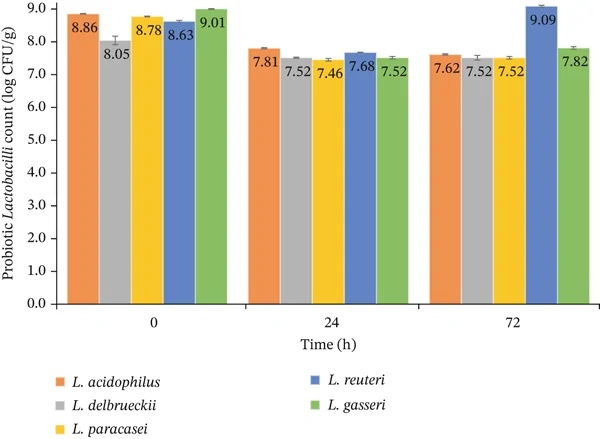
Growth pattern (log CFU/g) of different types of probiotic LAB spiked into AFM1‐contaminated BWC (7.5% brine) at 4°C.

**Figure 2:**
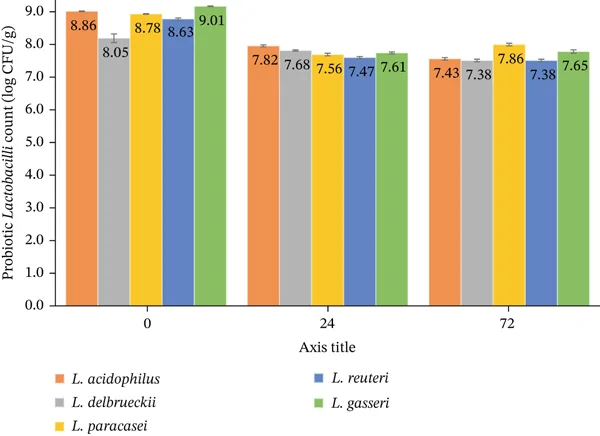
Growth pattern (log CFU/g) of different types of probiotic LAB spiked into AFM1‐contaminated BWC (7.5% brine) at 10°C.

**Figure 3:**
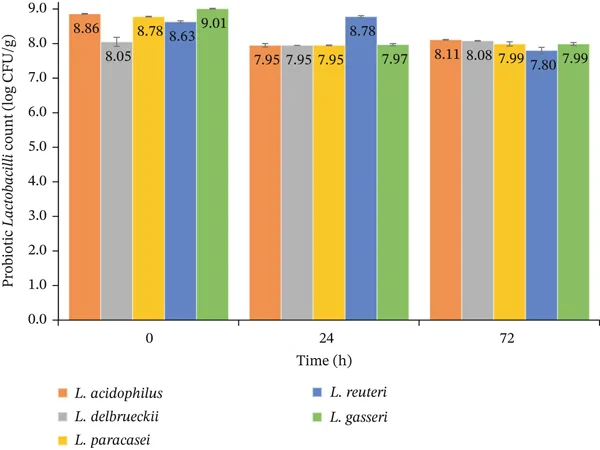
Growth pattern (log CFU/g) of different types of probiotic LAB spiked into AFM1‐contaminated BWC (7.5% brine) at 24°C.

The growth pattern of probiotic LAB (*L. acidophilus* DSMZ 9126, *L. delbrueckii* subsp. delbrueckii DSMZ 20074, *L. paracasei* subsp. paracasei DSMZ 202071, *L. reuteri* [SS730, S3608, CF2], and *L. gasseri* DSMZ 20243) inoculated into BWC (7.5% brine concentration) contaminated with AFM1 and stored for 72 h at 4°C, 10°C, or 24°C is presented in Figures 1, 2, and 3. The probiotic LAB was inoculated in BWC at an initial level of ca. 8.0–9.0 log CFU/g. In general, the counts of the probiotic LAB decreased slightly after incubation for all strains except for *L. reuteri* at 4°C, where the counts significantly increased from 8.63 to 9.09 log CFU/g compared with other probiotics. However, at 24°C, the counts of *L. delbrueckii* remained relatively stable. A reduction in the counts of probiotic strains was noticed at 4°C and 10°C after 72 h. *L. acidophilus*, *L. gasseri*, and *L. paracasei* exhibited slight but significant reductions (*p* < 0.05) in the counts compared with other probiotics at 4°C. Also, *L. acidophilus*, *L. gasseri*, and *L. reuteri* counts exhibited significant reduction at 10°C.

### 3.4. Detoxification of AFM1 From BWC by Different Types of Probiotic LAB at Different Storage Temperatures

**Figure 4:**
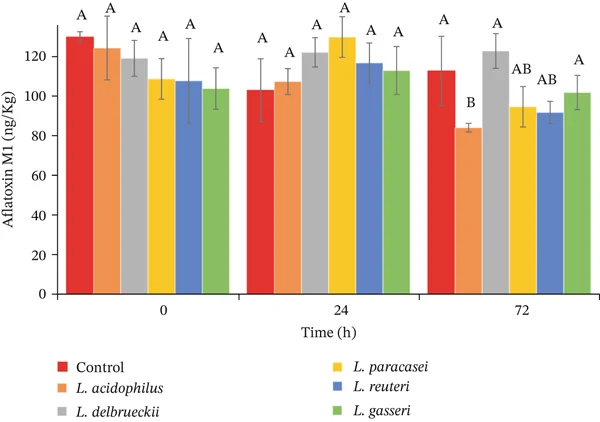
Detoxification of AFM1 (ng/kg) by different types of probiotic LAB inoculated into BWC (7.5% brine) at 4°C. Bars with different capital letters are significantly different (*p* < 0.05).

**Figure 5:**
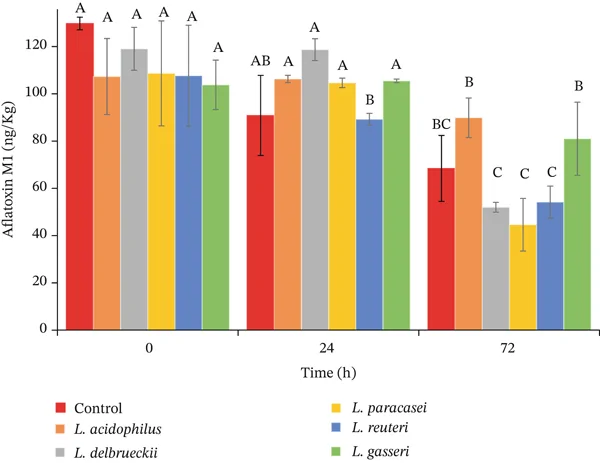
Detoxification of AFM1 (ng/kg) by different types of probiotic LAB inoculated into BWC (7.5% brine) at 10°C. Bars with different capital letters are significantly different (*p* < 0.05).

**Figure 6:**
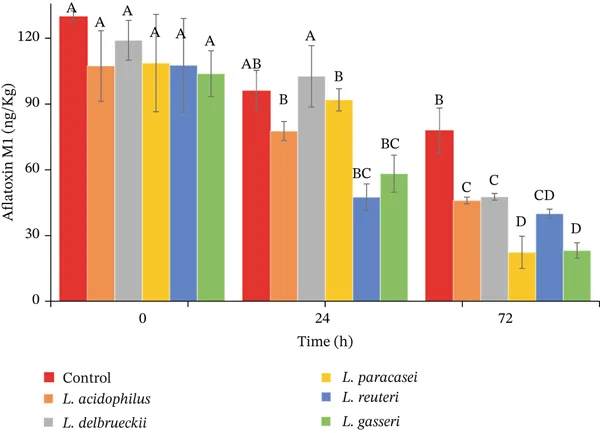
Detoxification of AFM1 (ng/kg) by different types of probiotic LAB inoculated into BWC (7.5% brine) at 24°C. Bars with different capital letters are significantly different (*p* < 0.05).

The ability of different strains of probiotic LAB to detoxify AFM1 from BWC is presented in Figures 4, 5, and 6. Five probiotic LAB (*L. acidophilus*, *L. delbrueckii*, *L. paracasei*, *L. reuteri*, and *L. gasseri*) were used and tested at three different temperatures (4°C, 10°C, and 24°C) for 0, 24, and 72 h. The initial level of AFM1 in BWC was ca. 125.0 ng/kg. The detoxification patterns were apparently dependent on the probiotics type, storage temperatures, and incubation durations. Generally, *L. paracasei*, *L. gasseri*, and *L. reuteri* were the most effective at reducing AFM1 levels compared with the control, *L. acidophilus* and *L. delbrueckii.* Evidently, the extent of reduction was more pronounced at 24°C (Figure 6) and increased as the incubation period increased to 72 h. *L. paracasei* diminished the levels of AFM1 from an initial level of 108 ng/kg to 44 and 22 ng/kg at 10°C, and 24°C, consecutively, after 72 h of incubation. All the probiotic strains exhibited minimal detoxification activity at 4°C (Figure 4). *L. reuteri* was also effective in eliminating AFM1 from BWC, where the AFM1 concentration significantly (*p* < 0.05) decreased from an initial level of 107 ng/kg to 53 and 40 ng/kg at 10°C and 24°C, consecutively, after 72 h of incubation.

## 4. Discussion

### 4.1. Changes in Salt Concentration, a_w_, and pH Values of BWC Inoculated With Different Strains of Probiotic Lactic Acid Bacteria

In the present study, white cheese was preserved into 7.5% brine solution. The initial salt concentration in the different cheese samples ranged from 3.18% to 3.27% (Table 1). However, the concentration of salt increased by the end of the incubation period to become in the range of 3.30%–3.72% and the higher salt concentrations were attained at the higher storage temperatures (10°C and 24°C), which were significantly higher than that at 4°C. The a_w_ values were in the range of 0.95–0.97. The a_w_ was lower at 24°C (0.95), yet this value was not significantly different than the a_w_ values at 4°C and 10°C.

### 4.2. Changes in pH Values of BWC Treated With Different Strains of Probiotic Lactic Acid Bacteria

As it is evident in Table 2, dramatic changes in the pH values occurred, especially in the BWC inoculated with the different types of probiotic LAB. The drop in pH values was unequivocally higher at high storage temperature (24°C). *L. paracasei* and *L. gasseri* elicited the highest reduction in pH values compared with the control and the rest of the probiotic LAB, especially at 10°C and 24°C. The drop in pH values is probably attributable to lactic acid production by the inoculated probiotic LAB, which could ferment lactose [32]. In Kareish cheese inoculated with ca. 2.0 × 10^7^ CFU/g *Bifidobacterium bifidum* DI and *L. rhamnosus* and stored at 5°C–7°C for 28 days, the initial pH was 5.3 and decreased slightly but non‐significantly to 5.0 by the end of the storage period [33]. In another study that investigated the physicochemical and microbiological properties of Kalari cheese, which is a famous cheese found in the Himalayan region, it was indicated that the pH of the cheese decreased significantly from 5.53 to 4.50 for the cheese stored under refrigeration. In comparison, no appreciable change in pH was observed for cheese stored at −18°C [34]. In a later study, soft cheese manufactured from goats’ milk and inoculated with the probiotics, *L. casei* and *B. longum*, and stored for 90 days at either 8°C or 13°C. The counts of the probiotic LAB decreased by almost 1 log by the end of 90‐day storage time, and the pH showed a slight increase from 4.6 to 4.9, regardless of the storage conditions [35]. Pasta filata cheeses were inoculated with one of two probiotic *Lactobacilli*: *L. rhamnosus* GG or *L. acidophilus* LA5, or their combination. The cheese was stored at 4°C or 12°C for 29 days. No changes in pH were observed in any of the probiotic cheese types stored at 4°C. However, *L. rhamnosus* was able to grow by 1.5 log at 12°C, while. *L. acidophilus* did not grow. Concomitantly, *L. rhamnosus* GG resulted in a considerable drop in the pH after 8 days of storage and till the end of the 29‐day storage period compared with *L. acidophilus* [36]. In another study, cheese was prepared from goats’ milk inoculated with a mixed culture of *L. plantarum* TW14 and *L. rhamnosus* TW2. The cheese was stored for 30 days at 4°C. The pH of the cheese dropped from an initial value of 4.78 at day zero to 4.42 after 30 days [37]. In agreement with the results obtained in the current study, white cheese samples were stored at either of 5°C, 15°C, or 25°C. A drop in pH occurred under all storage conditions, with the largest drop observed at higher storage temperatures, where pH values dropped from an initial value of 5.84 to 5.22 and 4.80 at 5°C and 24°C, respectively [38].

### 4.3. Growth Pattern of Probiotic LAB in BWC

In this study, the growth behavior of different types of probiotic LAB inoculated in BWC contaminated with AFM1 was studied at three different temperatures for 24 and 72 h of incubation. The initial level of inoculation was between 8.0 and 9.0 log CFU/g. At 4°C, all of the strains (*L. acidophilus*, *L. delbrueckii* subsp. delbrueckii, *L paracasei* subsp. paracasei, and *L. gasseri*) decreased slightly to ca. 7.60 log CFU/g after 72 h of incubation. However, for *L. reuteri*, the counts increased significantly to 9.09 log CFU/g toward the end of the incubation period (Figure 1). The same growth pattern was also observed at 10°C, where all strains decreased by ca. 1.0 log after 24 and 72 h of incubation (Figure 2). In contrast, at 24°C, all of the probiotic strains exhibited counts of ca. 8.0 log CFU/g after 72 h incubation (Figure 3). These results agree with those of Setyawardani et al. [39], who tracked the growth of some inoculated probiotic bacteria (*B. bifidum* and *Enterococcus faecium*) in probiotic UF white cheese for 60 days. The probiotic LAB counts remained stable at counts higher than 6.0 log CFU/g till the end of the 60‐day storage duration [40]. In another study, the viability of LAB (*L. casei*, *L. agilis*, *L. helveticus*, *L. delbrueckii*, *Streptococcus salivarius*, and *L. plantarum*) isolated from traditional cheeses and co‐cultured in Iranian white cheese was followed for a 60‐day ripening period. The initial level of inoculation was between 9.32 and 9.82 log CFU/g. After Day 3, all the strains showed a slight reduction to 8.8 log CFU/g except for *S. salivarius*, which exhibited a stable count. By the end of storage duration, *L. plantarum*, *L. casei*, and *L. helveticus* remained above 6.0 log CFU/g, while *L. agilis*, *L. delbrueckii*, and *L. salivarius* decreased to 5.52, 4.56, and 4.72 Log CFU/g, respectively [41].

To produce probiotic white cheese, *S. salivarius*, as a probiotic LAB was added along with other co‐cultures of LAB such as *L. rhamnosus*, *L. acidophilus*, and *L. casei* to pasteurized milk that had been cooled to 37°C. All the LAB bacteria were added at levels of ca. 9.0 log CFU/g of cheese and the survival of the probiotic bacteria was followed for 28 days of storage at 4°C. The probiotic bacteria decreased by ca. 1.0–2.0 logs after 1 week of storage. Nonetheless, the counts began to grow progressively until reaching ca. 9.5 log CFU/g toward the end of incubation time except for *L. acidophilus*, which decreased to ca. 7.5 log CFU/g [42].

### 4.4. Detoxification of AFM1 From BWC by Different Types of Probiotic Lactic Acid Bacteria

As shown in Figures 4, 5, and 6, the initial level of AFM1 in BWC was ca. 125.0 ng/kg. Five different strains of probiotic LAB (*L. acidophilus*, *L. delbrueckii*, *L. paracasei*, *L. reuteri*, and *L. gasseri*) were inoculated individually into the cheese at an approximate initial level of 8.0–9.0 log CFU/g (Figures 1, 2, and 3). There was an evident disparity in the abilities of the different LAB species to decontaminate AFM1 from BWC. Additionally, the detoxification efficacy was influenced by the storage temperature and incubation duration. The decontaminating efficiency of LAB species was more pronounced at higher storage temperature (24°C) and longer incubation period. *L. paracasei* was the most prominent in terms of its capability to bio‐remove AFM1 from BWC at 24°C. It resulted in reducing the level of the AFM1 from an initial level of ca. 125.0 ng/kg to 22.2 ng/kg, after 72 h of incubation at 24°C. Also, *L. gasseri* was similarly efficient, resulting in a reduction of AFM1 to ca. 23.0 ng/kg under the same storage conditions. Additionally, *L. reuteri*, resulted in reducing the concentration of AFM1 to 40.0 ng/kg after 72 h at 24°C. Although *L. acidophilus* and *L. delbrueckii* were not equally efficient as *L. paracasei*, *L reuteri*, and *L. gasseri*, they elicited significantly lower (*p* < 0.05) concentrations of AFM1 than the control. When the BWC samples were stored at 10°C for 72 h, both of *L. paracasei* and *L. reuteri* resulted in reducing the levels to 44.3 and 53.8 ng/kg, respectively, which were significantly lower (*p* < 0.05) compared with the control and the other probiotic LAB. However, when BWC was stored at 4°C, the detoxification capabilities of the different probiotics were generally less effective in diminishing AFM1. Yet *L. paracasei* and *L. reuteri* exerted significantly higher (*p* < 0.05) decontaminative activity of AFM1 after 72 h at 4°C compared with the control and other probiotics.

Several studies have examined the potential application of LAB as a biological detoxification agent [43]. *L. acidophilus* (La5) was examined for its detoxification activity of AFM1 in milk and it was able to reduce the concentration of the inoculated toxin from 75 to 28.2 ng/kg after 3 days under refrigeration [44]. In another more recent study to assess the potential of different strains of LAB to reduce the concentration of AFM1 that was artificially added to whole milk at the level of 50 ng/mL, *L. paracasei* was the most efficient among all examined LAB, where it resulted in ca. 70% reduction in the initial AFM1 concentration [30]. In another study, *L. acidophilus* DSMZ 20079, *L. rhamnosus* GG, and *B. bifidum* DSMZ 20456 were inoculated separately at the level 10^8^ CFU/mL of cows, sheep, or goat milk that were contaminated with AFM1 at a concentration of 100 ppt and then the inoculated milk samples were incubated for 4 h at 37°C followed by storage at 4°C for 24 h. The maximum AFM1 bio‐removal activity was achieved by *L. acidophilus* and *L. rhamnosus*, yet, the magnitude of reduction did not surpass 40% of the original inoculated AFM1 [31]. In another study, *L. reuteri* NRRL B‐14171, which was inoculated into AFM1 artificially contaminated milk, resulted in about 27% reduction of AFM1 [45]. In a later study evaluating the efficacy of *L. paracasei* KC39 and *L. plantarum* RM1 in reducing AFM1 in reconstituted milk powder, *L. paracasei* KC39 was more effective than *L. plantarum* RM1 [46]. However, Adácsi et al. [30] reported that *L. paracasei* subsp. paracasei was only able to reduce the initial level of AFM1 by 16%. In another study, *L. rhamnosus* and *L. lactis* were examined for their ability to decontaminate AFM1 from Minas Frescal cheese; the concentration of AFM1 decreased from 0.5 to 0.13 *μ*g/kg after 2 days of refrigerated storage compared with the untreated cheese [46].

The mechanism by which probiotic LAB detoxify AFM1 is not fully understood yet. However, it is postulated the main mechanism by which probiotic LAB undertake detoxification is by physical binding to peptidoglycans, polysaccharides, lipoteichoic, and teichoic acid [47–49]. AFM1 can bind to polysaccharides and peptidoglycans through noncovalent bonds, including hydrogen bonds, electrostatic, and van der Waals interactions [50]. Probiotic LAB were shown to exhibit a capability to eliminate mycotoxins, whether dead or viable cells are applied to an *in vivo* or *in vitro* system containing mycotoxins [51]. It was also suggested that organic acid produced by probiotic LAB could contribute to mycotoxin removal by deionizing mycotoxins and bio‐transforming them into less toxic metabolites [50]. Another possible way is potentially by degradation of aflatoxin through certain metabolic mechanism as it was reported that proteolytic enzymes of probiotic LAB could potentially detoxify mycotoxins [52, 53]. Biodegradation by bacterial extracellular proteins and enzymes could break the lactone ring of aflatoxin turning it into less toxic compounds [54, 55]. Increasing incubation temperature was found to increase enzymatic activity and biodegradation of aflatoxins by *Bacillus amyloliquefaciens* [55]. It was also postulated that probiotic LAB could downregulate the expression of gene (*aflR* gene) involved in the synthesis of aflatoxins [*56*]. In a later study, the probiotic *B. amyloliquefaciens* reduced aflatoxin B1 synthesis in *A. flavus* by suppressing the expression of 10 aflatoxin pathway genes and 2 transcription factors (alfR and alfS) [55]. It was also indicated that the extent of aflatoxins removal depends on several factors including the species of probiotic, experimental factors, including incubation period and time, food type, and the presence of prebiotics. The use of prebiotics that stimulate the growth of probiotic LAB such as *β*‐glucan and inulin could affect the detoxification efficacy of probiotic [57]. Notwithstanding, the extent of the detoxification process is bacterial dose‐dependent, and the quantity of mycotoxins binding is proportionally related to increasing probiotic cell concentration [58]. Although LABs are the most predominantly utilized probiotics to reduce AFM1, it was reported that the use of mixed probiotic strains, including bacteria or bacteria + yeast, demonstrated an improved reduction in the amount of aflatoxin in food. The combined use of probiotic bacteria (*B. bifidum*) and probiotic yeast (*Saccharomyces cerevisiae* ATCC9763) strains in milk resulted in a considerable reduction in the amount of AFM1 between 82.8% and 90% [50, 59].

## 5. Conclusions

BWC is a widely consumed cheese in the Eastern Mediterranean. Aflatoxins can enter the milk from contaminated animal feed and become part of the cheese during renneting or may develop from mold growth during storage and aging. In this study, various probiotic LAB strains were tested for their ability to detoxify AFM1 in BWC under different storage conditions and durations. These probiotic strains survived well across all tested temperatures. Notably, *L. paracasei* subsp. paracasei DSMZ 202071, *L. gasseri* DSMZ 20243, and *L. reuteri* (SS730, S3608, CF2) showed significant AFM1 detoxification activity, which was enhanced at higher temperature (24°C) and longer incubation (72 h). The findings indicate that *L. paracasei* subsp. paracasei and *L. reuteri* are particularly effective at reducing AFM1 in BWC. Further research is needed to assess additional LAB strains’ detoxifying potential in various dairy products. Additionally, exploring the use of probiotic LAB to inhibit the growth of bacteria and toxin production, such as toxins from *Staphylococcus aureus* and *Bacillus cereus*, as well as other mycotoxins, under different conditions like vacuum packaging and varying salt concentrations in BWC, is recommended.

## Author Contributions

The study conception and design, material preparation, data collection and analysis were performed by Hiba A. Al‐Masri, Murad A. Al‐Holy, and Amin N. Olaimat. Data analysis and revising initial draft were contributed by Murad A. Al‐Holy, Hiba A. Al‐Masri, and Amin N. Olaimat. Hamzah Al‐Qadiri, Maisa Monjed Monjed, Mutamed Ayyash, Mahmoud Abughoush, and Ma’mon M. Gharaibeh contributed to conceptualization, writing and final revisions of the manuscript.

## Funding

This project was financially supported by the Deanship of Scientific Research and Faculty of Graduate Studies at The Hashemite University, Zarqa, Jordan.

## Disclosure

All authors read and approved the final manuscript.

## Conflicts of Interest

The authors declare no conflicts of interest.

## Data Availability

The data that support the findings of this study are available from the corresponding author upon reasonable request.
